# 4β-Hy­droxy-12,13-ep­oxy­trichothec-9-ene

**DOI:** 10.1107/S1600536812012408

**Published:** 2012-03-28

**Authors:** Bi-Zeng Mao, Chao Huang, Yu-Zhe Chen, Zhen-Er Lv, Shao-Yuan Chen

**Affiliations:** a State Key Laboratory of Rice Biology and Key Laboratory of Molecular Biology of Crop Pathogens and Insects, Ministry of Agriculture, Institute of Biotechnology, Zhejiang University, Hangzhou, People’s Republic of China; b College of Pharmaceutical Science, Zhejiang University, People’s Republic of China

## Abstract

The asymmetric unit in the crystal of the title compound, C_15_H_22_O_3_, contains two independent mol­ecules with similar structures. Each mol­ecule contains two six-membered rings and one five-membered ring. The five-membered ring displays an envelope conformation with the C atom linking the epoxy group as the flap, while the two six-membered rings show half-chair conformations. The two independent mol­ecules are linked by an O—H⋯O hydrogen bond. These dimers are further linked by O—H⋯O hydrogen bonds, forming supra­molecular chains running along the *a* axis.

## Related literature

For the applications of trichodermin derivatives, see: Wei *et al.* (1974 ▶); Zhang *et al.* (2007 ▶). For ring conformations, see: Cremer & Pople (1975 ▶). For a related structure, see: Chen *et al.* (2008 ▶).
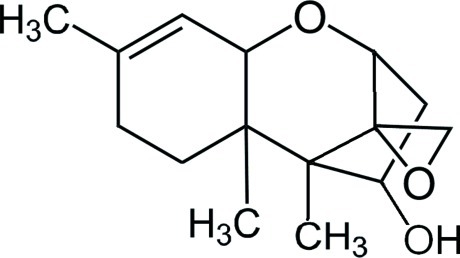


## Experimental

### 

#### Crystal data

C_15_H_22_O_3_
*M* *_r_* = 250.33
Orthorhombic, 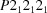

*a* = 7.0236 (5) Å
*b* = 12.0644 (10) Å
*c* = 32.475 (2) Å
*V* = 2751.8 (3) Å^3^
*Z* = 8
Mo *K*α radiation
μ = 0.08 mm^−1^
*T* = 296 K
0.52 × 0.38 × 0.10 mm

#### Data collection

Rigaku R-AXIS RAPID diffractometer
22591 measured reflections
2935 independent reflections
2054 reflections with *I* > 2σ(*I*)
*R*_int_ = 0.057

#### Refinement

*R*[*F* ^2^ > 2σ(*F* ^2^)] = 0.074
*wR*(*F* ^2^) = 0.222
*S* = 1.05
2935 reflections
333 parameters
18 restraints
H-atom parameters constrained
Δρ_max_ = 0.50 e Å^−3^
Δρ_min_ = −0.29 e Å^−3^

### 

Data collection: *PROCESS-AUTO* (Rigaku, 1998 ▶); cell refinement: *PROCESS-AUTO*; data reduction: *CrystalStructure* (Rigaku/MSC, 2005 ▶); program(s) used to solve structure: *SHELXS97* (Sheldrick, 2008 ▶); program(s) used to refine structure: *SHELXL97* (Sheldrick, 2008 ▶); molecular graphics: *ORTEP-3 for Windows* (Farrugia, 1997 ▶); software used to prepare material for publication: *WinGX* (Farrugia, 1999 ▶).

## Supplementary Material

Crystal structure: contains datablock(s) I, global. DOI: 10.1107/S1600536812012408/fj2530sup1.cif

Structure factors: contains datablock(s) I. DOI: 10.1107/S1600536812012408/fj2530Isup2.hkl

Additional supplementary materials:  crystallographic information; 3D view; checkCIF report

## Figures and Tables

**Table 1:** Hydrogen-bond geometry (Å, °)

*D*—H⋯*A*	*D*—H	H⋯*A*	*D*⋯*A*	*D*—H⋯*A*
O2*A*—H1*A*⋯O1*A*^i^	0.82	2.10	2.834 (4)	149
O2*B*—H1*B*⋯O2*A*	0.82	1.92	2.741 (6)	176

Symmetry code: (i) 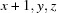.

**Fig. 1.**
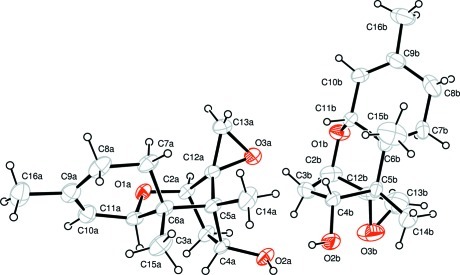
The molecular structure of (I) with 25% probability displacement ellipsoids (arbitrary spheres for H atoms).

**Fig. 2.**
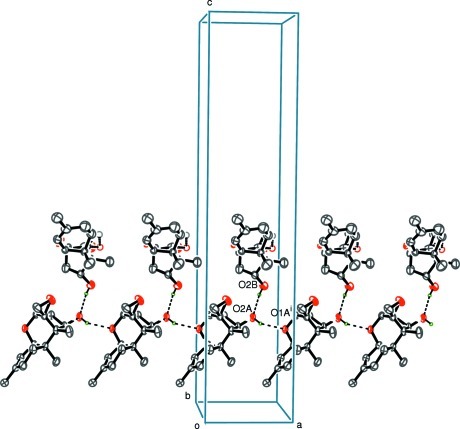
The crystal packing diagram showing hydrogen bonds (dashed lines).

## supplementary crystallographic information

### Comment

The title compound, deacetyl-trichodermin, is a trichodermin derivative. It shows potential bactericidal activity against Botrytis cinerea, Rhizoctonia solani and Pythium ultimum. Trichodermin is a member of the 4β-aceoxy-12,13-epoxytrichothecenes (Chen *et al.*, 2008), which form a medically and economically important class of mycotoxins produced by fungi that spoil fruit and grain (Zhang *et al.*, 2007). Many studies (Wei *et al.*, 1974) show that trichodermin is a very potent inhibitor of protein synthesis in mammalian cells. Trichodermin inhibits the elongation and/or termination processes of protein synthesis.

The assymetric unit of the title compound contains two independent molecules with the similar structure. The molecule contains two six membered rings and one five membered ring. The five membered ring displays an envelope conformation. In the C12A-containing ring the C12A atom lies at the flap position and is 0.680 (3) Å out of the mean plane formed by the other four atoms (Fig. 1).

Two six membered rings show distinct conformation. The O1A-containing ring displays a usual chair conformation. The ring puckering analysis for the C9A-containing six membered ring suggests a half-chair conformation (Cremer & Pople, 1975). The typical C9A═C10A double bond (Table 1) suggests these atoms are *sp*^2^ hybridized.

The two independent molecules are linked by an O—H···O hydrogen bond, forming the supra-molecular dimer (Table 1); the dimers are linked by O—H···O hydeogen bond to form the supra-molecular chains running along the *a* axis in the crystal (Fig. 2).

### Experimental

The title compound is prepared by deacetylation reaction of trichodermin in the presence of sodium hydroxide solution (3 *M*) at 333 K. The reaction miture was stirred for 30 min, then extracted with petroleum ether. The upper phase was filtered and evaporated *in vacuo*. Sample was then redissolved in petroleum ether to crystalize. The single crystlas were obtained by recrystalized from the hexane solution.

### Refinement

H atoms were placed in calculated positions with C—H = 0.93–0.98 Å, and O—H = 0.82 Å, and refined in riding mode with *U*_iso_(H) = 1.5*U*_eq_(C,*O*) for methyl H and hydroxy H atoms, and 1.2*U*_eq_(C) for the others. The O3b, C13*b* and C14B atoms are restrained so that their *U^ij^* components approximate to isotropic behavior. In the absence of significant anomalous scattering effects, Friedel pairs were merged, the absolute configuration was not determined.

### Crystal data

**Table:**

C_15_H_22_O_3_	*F*(000) = 1088
*M**_r_* = 250.33	*D*_x_ = 1.208 Mg m^−^^3^
Orthorhombic, *P*2_1_2_1_2_1_	Mo *K*α radiation, λ = 0.71073 Å
Hall symbol: P 2ac 2ab	Cell parameters from 15358 reflections
*a* = 7.0236 (5) Å	θ = 3.0–27.5°
*b* = 12.0644 (10) Å	µ = 0.08 mm^−^^1^
*c* = 32.475 (2) Å	*T* = 296 K
*V* = 2751.8 (3) Å^3^	Platelet, colourless
*Z* = 8	0.52 × 0.38 × 0.10 mm

### Data collection

**Table:**

Rigaku R-AXIS RAPID diffractometer	2054 reflections with *I* > 2σ(*I*)
Radiation source: rolling anode	*R*_int_ = 0.057
Graphite monochromator	θ_max_ = 25.5°, θ_min_ = 3.0°
Detector resolution: 10.00 pixels mm^-1^	*h* = −8→8
ω scans	*k* = −14→14
22591 measured reflections	*l* = −39→39
2935 independent reflections	

### Refinement

**Table:**

Refinement on *F*^2^	Primary atom site location: structure-invariant direct methods
Least-squares matrix: full	Secondary atom site location: difference Fourier map
*R*[*F*^2^ > 2σ(*F*^2^)] = 0.074	Hydrogen site location: inferred from neighbouring sites
*wR*(*F*^2^) = 0.222	H-atom parameters constrained
*S* = 1.05	*w* = 1/[σ^2^(*F*_o_^2^) + (0.1337*P*)^2^ + 0.8125*P*] where *P* = (*F*_o_^2^ + 2*F*_c_^2^)/3
2935 reflections	(Δ/σ)_max_ = 0.001
333 parameters	Δρ_max_ = 0.50 e Å^−^^3^
18 restraints	Δρ_min_ = −0.29 e Å^−^^3^

### Special details

**Table:**

Geometry. All e.s.d.'s (except the e.s.d. in the dihedral angle between two l.s. planes) are estimated using the full covariance matrix. The cell e.s.d.'s are taken into account individually in the estimation of e.s.d.'s in distances, angles and torsion angles; correlations between e.s.d.'s in cell parameters are only used when they are defined by crystal symmetry. An approximate (isotropic) treatment of cell e.s.d.'s is used for estimating e.s.d.'s involving l.s. planes.
Refinement. Refinement of *F*^2^ against ALL reflections. The weighted *R*-factor *wR* and goodness of fit *S* are based on *F*^2^, conventional *R*-factors *R* are based on *F*, with *F* set to zero for negative *F*^2^. The threshold expression of *F*^2^ > σ(*F*^2^) is used only for calculating *R*-factors(gt) *etc*. and is not relevant to the choice of reflections for refinement. *R*-factors based on *F*^2^ are statistically about twice as large as those based on *F*, and *R*- factors based on ALL data will be even larger.

### Fractional atomic coordinates and isotropic or equivalent isotropic displacement parameters (Å^2^)

**Table:**

	*x*	*y*	*z*	*U*_iso_*/*U*_eq_	
O1A	−0.0483 (4)	0.2711 (3)	0.22098 (10)	0.0508 (9)	
O2A	0.5717 (4)	0.2768 (4)	0.25029 (12)	0.0615 (10)	
H1A	0.6610	0.2602	0.2352	0.092*	
O3A	0.2507 (7)	0.1261 (4)	0.29715 (13)	0.0810 (14)	
C2A	0.0776 (7)	0.2677 (5)	0.25615 (16)	0.0524 (13)	
H2A	0.0085	0.2823	0.2818	0.063*	
C3A	0.2507 (7)	0.3429 (5)	0.25198 (18)	0.0546 (13)	
H3A1	0.3002	0.3622	0.2789	0.066*	
H3A2	0.2168	0.4107	0.2377	0.066*	
C4A	0.4007 (6)	0.2775 (4)	0.22718 (18)	0.0505 (12)	
H4A	0.4218	0.3128	0.2004	0.061*	
C5A	0.3112 (7)	0.1593 (4)	0.22112 (17)	0.0522 (12)	
C6A	0.1846 (7)	0.1609 (4)	0.18080 (16)	0.0510 (12)	
C7A	0.0549 (8)	0.0578 (5)	0.1773 (2)	0.0654 (16)	
H7A1	0.1325	−0.0087	0.1781	0.079*	
H7A2	−0.0316	0.0556	0.2005	0.079*	
C8A	−0.0589 (9)	0.0597 (6)	0.1376 (2)	0.0798 (19)	
H8A1	0.0233	0.0370	0.1152	0.096*	
H8A2	−0.1609	0.0058	0.1397	0.096*	
C9A	−0.1422 (8)	0.1687 (6)	0.12756 (18)	0.0692 (17)	
C10A	−0.0994 (8)	0.2585 (6)	0.14889 (17)	0.0634 (15)	
H10A	−0.1655	0.3235	0.1432	0.076*	
C11A	0.0499 (7)	0.2611 (4)	0.18179 (15)	0.0496 (12)	
H11A	0.1265	0.3281	0.1778	0.059*	
C12A	0.1732 (7)	0.1567 (5)	0.25720 (16)	0.0541 (12)	
C13A	0.0998 (11)	0.0591 (6)	0.2790 (2)	0.085 (2)	
H13A	−0.0263	0.0639	0.2910	0.102*	
H13B	0.1336	−0.0135	0.2683	0.102*	
C14A	0.4598 (9)	0.0678 (5)	0.2210 (3)	0.084 (2)	
H14A	0.5213	0.0650	0.2474	0.127*	
H14B	0.5528	0.0827	0.2001	0.127*	
H14C	0.3993	−0.0020	0.2156	0.127*	
C15A	0.3130 (9)	0.1675 (7)	0.1428 (2)	0.082 (2)	
H15A	0.2402	0.1935	0.1197	0.122*	
H15B	0.3632	0.0953	0.1368	0.122*	
H15C	0.4160	0.2178	0.1480	0.122*	
C16A	−0.2736 (10)	0.1729 (8)	0.08985 (18)	0.091 (2)	
H16A	−0.3542	0.2370	0.0916	0.137*	
H16B	−0.3508	0.1073	0.0891	0.137*	
H16C	−0.1981	0.1771	0.0653	0.137*	
O1B	0.3469 (6)	0.2007 (4)	0.43814 (14)	0.0782 (13)	
O2B	0.6999 (7)	0.2956 (5)	0.32971 (13)	0.0941 (16)	
H1B	0.6615	0.2865	0.3061	0.141*	
O3B	0.7218 (12)	0.4044 (6)	0.42104 (19)	0.133 (2)	
C2B	0.4209 (10)	0.2993 (6)	0.4174 (2)	0.084 (2)	
H2B	0.3637	0.3674	0.4282	0.101*	
C3B	0.4153 (10)	0.2959 (7)	0.3716 (2)	0.092 (2)	
H3B1	0.2993	0.2601	0.3623	0.110*	
H3B2	0.4187	0.3705	0.3605	0.110*	
C4B	0.5840 (8)	0.2323 (6)	0.35772 (17)	0.0691 (17)	
H4B	0.5430	0.1634	0.3444	0.083*	
C5B	0.7004 (7)	0.2051 (6)	0.39682 (16)	0.0596 (14)	
C6B	0.6248 (7)	0.0929 (5)	0.41599 (17)	0.0573 (14)	
C7B	0.7008 (10)	0.0739 (8)	0.4601 (2)	0.098 (3)	
H7B1	0.8389	0.0724	0.4594	0.118*	
H7B2	0.6625	0.1356	0.4774	0.118*	
C8B	0.6291 (14)	−0.0325 (10)	0.4789 (3)	0.134 (4)	
H8B1	0.6950	−0.0944	0.4663	0.160*	
H8B2	0.6596	−0.0329	0.5081	0.160*	
C9B	0.4207 (12)	−0.0483 (6)	0.4739 (2)	0.083 (2)	
C10B	0.3238 (10)	0.0099 (5)	0.44710 (19)	0.0696 (16)	
H10B	0.1947	−0.0054	0.4443	0.084*	
C11B	0.4075 (7)	0.1008 (5)	0.42018 (17)	0.0545 (13)	
H11B	0.3511	0.0951	0.3927	0.065*	
C12B	0.6341 (14)	0.2953 (6)	0.4252 (2)	0.093 (3)	
C13B	0.7293 (19)	0.3454 (9)	0.4596 (3)	0.136 (4)	
H13C	0.8508	0.3145	0.4679	0.163*	
H13D	0.6511	0.3728	0.4820	0.163*	
C14B	0.9136 (10)	0.2066 (9)	0.3893 (3)	0.103 (2)	
H14D	0.9477	0.2735	0.3751	0.154*	
H14E	0.9485	0.1437	0.3728	0.154*	
H14F	0.9793	0.2034	0.4152	0.154*	
C15B	0.6810 (16)	−0.0047 (7)	0.3889 (3)	0.126 (4)	
H15D	0.6592	−0.0727	0.4035	0.189*	
H15E	0.8135	0.0010	0.3819	0.189*	
H15F	0.6060	−0.0040	0.3642	0.189*	
C16B	0.3229 (17)	−0.1340 (7)	0.5011 (2)	0.117 (3)	
H16D	0.3350	−0.1126	0.5294	0.176*	
H16E	0.3816	−0.2051	0.4970	0.176*	
H16F	0.1906	−0.1384	0.4939	0.176*	

### Atomic displacement parameters (Å^2^)

**Table:**

	*U*^11^	*U*^22^	*U*^33^	*U*^12^	*U*^13^	*U*^23^
O1A	0.0345 (16)	0.051 (2)	0.066 (2)	0.0018 (15)	0.0006 (16)	−0.0087 (17)
O2A	0.0352 (16)	0.066 (3)	0.083 (2)	−0.0061 (18)	0.0035 (19)	−0.008 (2)
O3A	0.078 (3)	0.077 (3)	0.088 (3)	−0.027 (2)	−0.027 (3)	0.021 (2)
C2A	0.038 (2)	0.054 (3)	0.065 (3)	−0.007 (2)	0.003 (2)	−0.014 (2)
C3A	0.041 (2)	0.039 (3)	0.085 (3)	0.002 (2)	−0.002 (3)	−0.012 (3)
C4A	0.031 (2)	0.041 (3)	0.079 (3)	0.001 (2)	0.002 (2)	−0.006 (3)
C5A	0.036 (2)	0.033 (2)	0.088 (3)	0.000 (2)	0.004 (3)	−0.005 (3)
C6A	0.038 (2)	0.044 (3)	0.071 (3)	0.000 (2)	0.010 (2)	−0.009 (3)
C7A	0.053 (3)	0.043 (3)	0.101 (4)	−0.007 (3)	0.001 (3)	−0.019 (3)
C8A	0.061 (3)	0.088 (5)	0.090 (4)	−0.018 (4)	0.005 (4)	−0.033 (4)
C9A	0.055 (3)	0.093 (5)	0.059 (3)	−0.007 (3)	0.012 (3)	−0.011 (3)
C10A	0.052 (3)	0.072 (4)	0.066 (3)	0.001 (3)	0.006 (3)	−0.003 (3)
C11A	0.042 (2)	0.045 (3)	0.061 (3)	−0.002 (2)	0.011 (2)	−0.002 (2)
C12A	0.044 (2)	0.048 (3)	0.070 (3)	−0.008 (3)	−0.002 (3)	0.007 (3)
C13A	0.088 (4)	0.079 (4)	0.089 (4)	−0.038 (4)	−0.021 (4)	0.021 (4)
C14A	0.056 (3)	0.049 (3)	0.148 (6)	0.019 (3)	−0.014 (4)	−0.021 (4)
C15A	0.052 (3)	0.098 (5)	0.094 (4)	−0.008 (4)	0.020 (3)	−0.035 (4)
C16A	0.061 (4)	0.149 (7)	0.064 (3)	−0.002 (4)	0.005 (3)	−0.011 (4)
O1B	0.073 (3)	0.063 (3)	0.099 (3)	0.011 (2)	0.037 (2)	0.010 (2)
O2B	0.072 (3)	0.138 (5)	0.072 (2)	−0.031 (3)	−0.001 (2)	0.034 (3)
O3B	0.161 (4)	0.117 (4)	0.120 (4)	−0.066 (4)	0.010 (4)	−0.002 (3)
C2B	0.082 (4)	0.053 (4)	0.117 (6)	0.007 (3)	0.039 (4)	−0.003 (4)
C3B	0.067 (4)	0.103 (6)	0.106 (5)	0.005 (4)	0.011 (4)	0.047 (5)
C4B	0.051 (3)	0.095 (5)	0.062 (3)	−0.016 (3)	−0.004 (3)	0.019 (3)
C5B	0.041 (3)	0.084 (4)	0.054 (3)	−0.002 (3)	−0.008 (2)	0.010 (3)
C6B	0.047 (3)	0.061 (3)	0.064 (3)	0.011 (3)	0.003 (3)	0.002 (3)
C7B	0.059 (4)	0.136 (7)	0.100 (5)	−0.004 (4)	−0.021 (4)	0.058 (5)
C8B	0.116 (7)	0.142 (9)	0.143 (8)	0.019 (7)	−0.015 (7)	0.078 (7)
C9B	0.105 (5)	0.065 (4)	0.078 (4)	−0.007 (4)	−0.002 (4)	0.022 (3)
C10B	0.068 (4)	0.063 (4)	0.078 (4)	−0.019 (3)	−0.003 (3)	0.006 (3)
C11B	0.042 (3)	0.059 (3)	0.062 (3)	−0.004 (2)	−0.002 (3)	0.002 (3)
C12B	0.140 (7)	0.074 (5)	0.066 (4)	−0.058 (5)	0.013 (4)	−0.005 (3)
C13B	0.155 (6)	0.133 (5)	0.118 (5)	−0.030 (5)	−0.003 (4)	−0.004 (4)
C14B	0.068 (3)	0.129 (5)	0.112 (4)	−0.002 (4)	−0.002 (3)	0.022 (4)
C15B	0.156 (9)	0.080 (5)	0.143 (7)	0.028 (6)	0.061 (7)	−0.006 (5)
C16B	0.159 (9)	0.090 (6)	0.103 (5)	−0.030 (6)	0.003 (6)	0.033 (5)

### Geometric parameters (Å, º)

**Table:**

O1A—C2A	1.445 (6)	O1B—C11B	1.405 (7)
O1A—C11A	1.452 (6)	O1B—C2B	1.462 (8)
O2A—C4A	1.417 (6)	O2B—C4B	1.440 (7)
O2A—H1A	0.8200	O2B—H1B	0.8200
O3A—C12A	1.454 (6)	O3B—C13B	1.441 (11)
O3A—C13A	1.458 (7)	O3B—C12B	1.459 (9)
C2A—C12A	1.499 (8)	C2B—C3B	1.487 (11)
C2A—C3A	1.523 (7)	C2B—C12B	1.520 (12)
C2A—H2A	0.9800	C2B—H2B	0.9800
C3A—C4A	1.543 (7)	C3B—C4B	1.483 (10)
C3A—H3A1	0.9700	C3B—H3B1	0.9700
C3A—H3A2	0.9700	C3B—H3B2	0.9700
C4A—C5A	1.571 (7)	C4B—C5B	1.545 (7)
C4A—H4A	0.9800	C4B—H4B	0.9800
C5A—C14A	1.519 (7)	C5B—C12B	1.500 (10)
C5A—C12A	1.521 (7)	C5B—C14B	1.517 (9)
C5A—C6A	1.583 (8)	C5B—C6B	1.582 (8)
C6A—C15A	1.531 (8)	C6B—C15B	1.522 (10)
C6A—C11A	1.535 (7)	C6B—C11B	1.535 (7)
C6A—C7A	1.547 (7)	C6B—C7B	1.547 (9)
C7A—C8A	1.515 (9)	C7B—C8B	1.509 (12)
C7A—H7A1	0.9700	C7B—H7B1	0.9700
C7A—H7A2	0.9700	C7B—H7B2	0.9700
C8A—C9A	1.476 (10)	C8B—C9B	1.485 (13)
C8A—H8A1	0.9700	C8B—H8B1	0.9700
C8A—H8A2	0.9700	C8B—H8B2	0.9700
C9A—C10A	1.320 (9)	C9B—C10B	1.309 (9)
C9A—C16A	1.534 (8)	C9B—C16B	1.523 (10)
C10A—C11A	1.497 (7)	C10B—C11B	1.521 (8)
C10A—H10A	0.9300	C10B—H10B	0.9300
C11A—H11A	0.9800	C11B—H11B	0.9800
C12A—C13A	1.467 (8)	C12B—C13B	1.435 (12)
C13A—H13A	0.9700	C13B—H13C	0.9700
C13A—H13B	0.9700	C13B—H13D	0.9700
C14A—H14A	0.9600	C14B—H14D	0.9600
C14A—H14B	0.9600	C14B—H14E	0.9600
C14A—H14C	0.9600	C14B—H14F	0.9600
C15A—H15A	0.9600	C15B—H15D	0.9600
C15A—H15B	0.9600	C15B—H15E	0.9600
C15A—H15C	0.9600	C15B—H15F	0.9600
C16A—H16A	0.9600	C16B—H16D	0.9600
C16A—H16B	0.9600	C16B—H16E	0.9600
C16A—H16C	0.9600	C16B—H16F	0.9600
			
C2A—O1A—C11A	113.6 (3)	C11B—O1B—C2B	113.5 (4)
C4A—O2A—H1A	109.5	C4B—O2B—H1B	109.5
C12A—O3A—C13A	60.5 (4)	C13B—O3B—C12B	59.3 (6)
O1A—C2A—C12A	108.5 (4)	O1B—C2B—C3B	115.4 (6)
O1A—C2A—C3A	113.7 (4)	O1B—C2B—C12B	104.3 (6)
C12A—C2A—C3A	100.2 (4)	C3B—C2B—C12B	101.1 (5)
O1A—C2A—H2A	111.3	O1B—C2B—H2B	111.8
C12A—C2A—H2A	111.3	C3B—C2B—H2B	111.8
C3A—C2A—H2A	111.3	C12B—C2B—H2B	111.8
C2A—C3A—C4A	106.6 (4)	C4B—C3B—C2B	107.3 (6)
C2A—C3A—H3A1	110.4	C4B—C3B—H3B1	110.3
C4A—C3A—H3A1	110.4	C2B—C3B—H3B1	110.3
C2A—C3A—H3A2	110.4	C4B—C3B—H3B2	110.3
C4A—C3A—H3A2	110.4	C2B—C3B—H3B2	110.3
H3A1—C3A—H3A2	108.6	H3B1—C3B—H3B2	108.5
O2A—C4A—C3A	107.8 (4)	O2B—C4B—C3B	111.7 (6)
O2A—C4A—C5A	113.6 (4)	O2B—C4B—C5B	109.4 (4)
C3A—C4A—C5A	104.9 (4)	C3B—C4B—C5B	106.4 (5)
O2A—C4A—H4A	110.1	O2B—C4B—H4B	109.7
C3A—C4A—H4A	110.1	C3B—C4B—H4B	109.7
C5A—C4A—H4A	110.1	C5B—C4B—H4B	109.7
C14A—C5A—C12A	115.1 (5)	C12B—C5B—C14B	113.4 (7)
C14A—C5A—C4A	112.6 (4)	C12B—C5B—C4B	100.8 (6)
C12A—C5A—C4A	100.2 (4)	C14B—C5B—C4B	112.8 (5)
C14A—C5A—C6A	113.2 (5)	C12B—C5B—C6B	106.0 (4)
C12A—C5A—C6A	106.2 (4)	C14B—C5B—C6B	113.9 (6)
C4A—C5A—C6A	108.5 (4)	C4B—C5B—C6B	109.1 (5)
C15A—C6A—C11A	109.8 (5)	C15B—C6B—C11B	111.0 (6)
C15A—C6A—C7A	109.2 (5)	C15B—C6B—C7B	109.4 (6)
C11A—C6A—C7A	105.8 (4)	C11B—C6B—C7B	105.7 (5)
C15A—C6A—C5A	109.7 (4)	C15B—C6B—C5B	110.3 (5)
C11A—C6A—C5A	109.8 (4)	C11B—C6B—C5B	108.4 (5)
C7A—C6A—C5A	112.5 (5)	C7B—C6B—C5B	112.0 (5)
C8A—C7A—C6A	111.2 (5)	C8B—C7B—C6B	112.7 (7)
C8A—C7A—H7A1	109.4	C8B—C7B—H7B1	109.1
C6A—C7A—H7A1	109.4	C6B—C7B—H7B1	109.1
C8A—C7A—H7A2	109.4	C8B—C7B—H7B2	109.1
C6A—C7A—H7A2	109.4	C6B—C7B—H7B2	109.1
H7A1—C7A—H7A2	108.0	H7B1—C7B—H7B2	107.8
C9A—C8A—C7A	114.3 (5)	C9B—C8B—C7B	113.2 (7)
C9A—C8A—H8A1	108.7	C9B—C8B—H8B1	108.9
C7A—C8A—H8A1	108.7	C7B—C8B—H8B1	108.9
C9A—C8A—H8A2	108.7	C9B—C8B—H8B2	108.9
C7A—C8A—H8A2	108.7	C7B—C8B—H8B2	108.9
H8A1—C8A—H8A2	107.6	H8B1—C8B—H8B2	107.8
C10A—C9A—C8A	121.6 (6)	C10B—C9B—C8B	121.1 (7)
C10A—C9A—C16A	121.9 (7)	C10B—C9B—C16B	121.0 (8)
C8A—C9A—C16A	116.4 (6)	C8B—C9B—C16B	117.9 (7)
C9A—C10A—C11A	123.5 (6)	C9B—C10B—C11B	124.6 (6)
C9A—C10A—H10A	118.3	C9B—C10B—H10B	117.7
C11A—C10A—H10A	118.3	C11B—C10B—H10B	117.7
O1A—C11A—C10A	107.1 (4)	O1B—C11B—C10B	105.3 (4)
O1A—C11A—C6A	112.1 (4)	O1B—C11B—C6B	113.0 (5)
C10A—C11A—C6A	113.6 (4)	C10B—C11B—C6B	113.0 (5)
O1A—C11A—H11A	107.9	O1B—C11B—H11B	108.5
C10A—C11A—H11A	107.9	C10B—C11B—H11B	108.5
C6A—C11A—H11A	107.9	C6B—C11B—H11B	108.5
O3A—C12A—C13A	59.9 (4)	C13B—C12B—O3B	59.7 (5)
O3A—C12A—C2A	114.5 (5)	C13B—C12B—C5B	129.7 (9)
C13A—C12A—C2A	124.8 (5)	O3B—C12B—C5B	117.8 (6)
O3A—C12A—C5A	117.0 (4)	C13B—C12B—C2B	125.1 (9)
C13A—C12A—C5A	127.7 (5)	O3B—C12B—C2B	111.8 (8)
C2A—C12A—C5A	104.5 (4)	C5B—C12B—C2B	103.1 (6)
O3A—C13A—C12A	59.6 (4)	C12B—C13B—O3B	61.0 (6)
O3A—C13A—H13A	117.8	C12B—C13B—H13C	117.7
C12A—C13A—H13A	117.8	O3B—C13B—H13C	117.7
O3A—C13A—H13B	117.8	C12B—C13B—H13D	117.7
C12A—C13A—H13B	117.8	O3B—C13B—H13D	117.7
H13A—C13A—H13B	114.9	H13C—C13B—H13D	114.8
C5A—C14A—H14A	109.5	C5B—C14B—H14D	109.5
C5A—C14A—H14B	109.5	C5B—C14B—H14E	109.5
H14A—C14A—H14B	109.5	H14D—C14B—H14E	109.5
C5A—C14A—H14C	109.5	C5B—C14B—H14F	109.5
H14A—C14A—H14C	109.5	H14D—C14B—H14F	109.5
H14B—C14A—H14C	109.5	H14E—C14B—H14F	109.5
C6A—C15A—H15A	109.5	C6B—C15B—H15D	109.5
C6A—C15A—H15B	109.5	C6B—C15B—H15E	109.5
H15A—C15A—H15B	109.5	H15D—C15B—H15E	109.5
C6A—C15A—H15C	109.5	C6B—C15B—H15F	109.5
H15A—C15A—H15C	109.5	H15D—C15B—H15F	109.5
H15B—C15A—H15C	109.5	H15E—C15B—H15F	109.5
C9A—C16A—H16A	109.5	C9B—C16B—H16D	109.5
C9A—C16A—H16B	109.5	C9B—C16B—H16E	109.5
H16A—C16A—H16B	109.5	H16D—C16B—H16E	109.5
C9A—C16A—H16C	109.5	C9B—C16B—H16F	109.5
H16A—C16A—H16C	109.5	H16D—C16B—H16F	109.5
H16B—C16A—H16C	109.5	H16E—C16B—H16F	109.5
			
C11A—O1A—C2A—C12A	−63.0 (5)	C11B—O1B—C2B—C3B	43.5 (7)
C11A—O1A—C2A—C3A	47.5 (5)	C11B—O1B—C2B—C12B	−66.4 (7)
O1A—C2A—C3A—C4A	−83.8 (5)	O1B—C2B—C3B—C4B	−81.8 (7)
C12A—C2A—C3A—C4A	31.8 (5)	C12B—C2B—C3B—C4B	30.1 (8)
C2A—C3A—C4A—O2A	−126.2 (5)	C2B—C3B—C4B—O2B	−123.7 (6)
C2A—C3A—C4A—C5A	−4.9 (6)	C2B—C3B—C4B—C5B	−4.3 (8)
O2A—C4A—C5A—C14A	−28.9 (7)	O2B—C4B—C5B—C12B	97.2 (6)
C3A—C4A—C5A—C14A	−146.4 (5)	C3B—C4B—C5B—C12B	−23.6 (6)
O2A—C4A—C5A—C12A	93.9 (5)	O2B—C4B—C5B—C14B	−24.0 (9)
C3A—C4A—C5A—C12A	−23.5 (5)	C3B—C4B—C5B—C14B	−144.8 (7)
O2A—C4A—C5A—C6A	−155.0 (4)	O2B—C4B—C5B—C6B	−151.5 (5)
C3A—C4A—C5A—C6A	87.5 (5)	C3B—C4B—C5B—C6B	87.6 (6)
C14A—C5A—C6A—C15A	−54.6 (6)	C12B—C5B—C6B—C15B	178.8 (7)
C12A—C5A—C6A—C15A	178.1 (5)	C14B—C5B—C6B—C15B	−55.8 (8)
C4A—C5A—C6A—C15A	71.2 (5)	C4B—C5B—C6B—C15B	71.1 (7)
C14A—C5A—C6A—C11A	−175.4 (5)	C12B—C5B—C6B—C11B	57.1 (6)
C12A—C5A—C6A—C11A	57.3 (5)	C14B—C5B—C6B—C11B	−177.5 (6)
C4A—C5A—C6A—C11A	−49.6 (5)	C4B—C5B—C6B—C11B	−50.6 (6)
C14A—C5A—C6A—C7A	67.1 (6)	C12B—C5B—C6B—C7B	−59.1 (7)
C12A—C5A—C6A—C7A	−60.2 (5)	C14B—C5B—C6B—C7B	66.3 (8)
C4A—C5A—C6A—C7A	−167.1 (4)	C4B—C5B—C6B—C7B	−166.8 (5)
C15A—C6A—C7A—C8A	−55.9 (6)	C15B—C6B—C7B—C8B	−57.6 (9)
C11A—C6A—C7A—C8A	62.2 (6)	C11B—C6B—C7B—C8B	61.9 (9)
C5A—C6A—C7A—C8A	−177.9 (5)	C5B—C6B—C7B—C8B	179.7 (7)
C6A—C7A—C8A—C9A	−43.6 (7)	C6B—C7B—C8B—C9B	−47.3 (12)
C7A—C8A—C9A—C10A	8.0 (9)	C7B—C8B—C9B—C10B	16.2 (15)
C7A—C8A—C9A—C16A	−174.7 (5)	C7B—C8B—C9B—C16B	−163.5 (8)
C8A—C9A—C10A—C11A	6.6 (9)	C8B—C9B—C10B—C11B	−2.7 (13)
C16A—C9A—C10A—C11A	−170.6 (5)	C16B—C9B—C10B—C11B	177.0 (7)
C2A—O1A—C11A—C10A	176.3 (4)	C2B—O1B—C11B—C10B	177.5 (6)
C2A—O1A—C11A—C6A	51.1 (5)	C2B—O1B—C11B—C6B	53.7 (7)
C9A—C10A—C11A—O1A	−108.8 (6)	C9B—C10B—C11B—O1B	−104.0 (7)
C9A—C10A—C11A—C6A	15.5 (7)	C9B—C10B—C11B—C6B	19.8 (9)
C15A—C6A—C11A—O1A	−168.6 (4)	C15B—C6B—C11B—O1B	−168.6 (5)
C7A—C6A—C11A—O1A	73.7 (5)	C7B—C6B—C11B—O1B	73.0 (7)
C5A—C6A—C11A—O1A	−47.9 (5)	C5B—C6B—C11B—O1B	−47.2 (6)
C15A—C6A—C11A—C10A	69.8 (6)	C15B—C6B—C11B—C10B	72.0 (7)
C7A—C6A—C11A—C10A	−47.9 (6)	C7B—C6B—C11B—C10B	−46.4 (7)
C5A—C6A—C11A—C10A	−169.5 (4)	C5B—C6B—C11B—C10B	−166.7 (4)
C13A—O3A—C12A—C2A	117.5 (6)	C13B—O3B—C12B—C5B	−121.9 (11)
C13A—O3A—C12A—C5A	−119.8 (6)	C13B—O3B—C12B—C2B	119.0 (9)
O1A—C2A—C12A—O3A	−159.7 (4)	C14B—C5B—C12B—C13B	−32.9 (10)
C3A—C2A—C12A—O3A	80.9 (5)	C4B—C5B—C12B—C13B	−153.6 (8)
O1A—C2A—C12A—C13A	−90.5 (6)	C6B—C5B—C12B—C13B	92.7 (9)
C3A—C2A—C12A—C13A	150.1 (5)	C14B—C5B—C12B—O3B	39.5 (10)
O1A—C2A—C12A—C5A	71.0 (5)	C4B—C5B—C12B—O3B	−81.3 (8)
C3A—C2A—C12A—C5A	−48.3 (5)	C6B—C5B—C12B—O3B	165.1 (7)
C14A—C5A—C12A—O3A	38.4 (7)	C14B—C5B—C12B—C2B	163.1 (6)
C4A—C5A—C12A—O3A	−82.7 (5)	C4B—C5B—C12B—C2B	42.3 (6)
C6A—C5A—C12A—O3A	164.5 (4)	C6B—C5B—C12B—C2B	−71.3 (6)
C14A—C5A—C12A—C13A	−33.1 (8)	O1B—C2B—C12B—C13B	−90.5 (9)
C4A—C5A—C12A—C13A	−154.1 (5)	C3B—C2B—C12B—C13B	149.4 (8)
C6A—C5A—C12A—C13A	93.1 (6)	O1B—C2B—C12B—O3B	−158.0 (5)
C14A—C5A—C12A—C2A	166.1 (5)	C3B—C2B—C12B—O3B	81.9 (7)
C4A—C5A—C12A—C2A	45.0 (5)	O1B—C2B—C12B—C5B	74.5 (6)
C6A—C5A—C12A—C2A	−67.8 (5)	C3B—C2B—C12B—C5B	−45.6 (7)
C2A—C12A—C13A—O3A	−100.4 (6)	C5B—C12B—C13B—O3B	102.5 (9)
C5A—C12A—C13A—O3A	102.4 (6)	C2B—C12B—C13B—O3B	−96.6 (9)

### Hydrogen-bond geometry (Å, º)

**Table:**

*D*—H···*A*	*D*—H	H···*A*	*D*···*A*	*D*—H···*A*
O2*A*—H1*A*···O1*A*^i^	0.82	2.10	2.834 (4)	149
O2*B*—H1*B*···O2*A*	0.82	1.92	2.741 (6)	176

Symmetry code: (i) *x*+1, *y*, *z*.
